# Atorvastatin reduces alloxan-induced impairment of aversive stimulus memory in mice

**DOI:** 10.2478/abm-2022-0009

**Published:** 2022-04-29

**Authors:** Osman Kukula, Caner Günaydın

**Affiliations:** Department of Pharmacology, Ondokuz Mayıs University, Faculty of Medicine, Atakum, Samsun 55139, Turkey

**Keywords:** alloxan, atorvastatin, avoidance learning, diabetes mellitus, experimental, memory

## Abstract

**Background:**

An association between dysregulated glucose levels in patients with diabetes mellitus and detrimental effects on the central nervous system, particularly in Alzheimer disease, has been recognized. Atorvastatin treatment has improved memory and cognition in some patients with diabetes mellitus and Alzheimer disease.

**Objectives:**

To determine possible neuroprotective effects of atorvastatin on memory and cognition by measuring changes in an adverse stimulus avoidance learning deficit induced by alloxan in a murine model of diabetes mellitus and impaired memory and cognition.

**Methods:**

We administered 150 mg/kg and 100 mg/kg alloxan in saline (intraperitoneally, i.p.) at a 48 h interval to produce a model of diabetes mellitus in male BALB/c mice. An oral glucose tolerance test (OGTT) was used to assess blood glucose regulation. After demonstrating hyperglycemia in mice (n = 7 per group) we administered vehicle (saline, i.p.), atorvastatin (10 mg/kg, i.p.), or liraglutide (200 μg/kg, i.p.) for 28 d except for those in a negative control group, which were given saline instead of alloxan, and a group administered atorvastatin alone, which were given saline instead of alloxan followed by atorvastatin (10 mg/kg, i.p.) for 28 d. Locomotor activity was measured 24 h after the final drug treatments, and subsequently their learned behavioral response to an adverse electrical stimulus to their plantar paw surface in a dark compartment was measured using a passive avoidance apparatus (Ugo Basile) in a model of impaired memory and cognition associated with Alzheimer disease. To determine any deficit in their learned avoidance of the adverse stimulus, we measured the initial latency or time mice spent in an illuminated white compartment before entering the dark compartment in the learning trial, and on the day after learning to avoid the adverse stimulus, the retention period latency in the light compartment and time spent in the dark compartment.

**Results:**

Atorvastatin alone produced no significant change in blood glucose levels (*F*_4,10_ = 0.80, *P* = 0.55) within 2 h. Liraglutide decreased blood glucose levels after 0.5 h (*F*_4,10_ = 11.7, *P* < 0.001). We found no significant change in locomotor activity in any group. In mice with alloxan-induced diabetes, atorvastatin significantly attenuated the decreased avoidance associated with the diabetes (*F*_4,30_ = 38.0, *P* = 0.02) and liraglutide also significantly attenuated the decreased avoidance (*F*_4,30_ = 38.0, *P* < 0.001). Atorvastatin alone had no significant effect on the adversive learned response compared with vehicle treatment (*F*_4,30_ = 38.0, *P* > 0.05). Atorvastatin significantly decreased the time mice with alloxan-induced diabetes spent in the dark compartment compared with mice in the diabetes group without atorvastatin treatment (*F*_4,30_ = 53.9, *P* = 0.046). Liraglutide also significantly reduced the time mice with alloxan-induced diabetes spent in the dark compartment compared with vehicle-treated mice with alloxan-induced diabetes (*F*_4,30_ = 53.9, *P* < 0.001). Atorvastatin treatment alone had no significant effect on the time mice spent in dark compartment compared with the control group (*F*_4,30_ = 53.9, *P* > 0.05).

**Conclusion:**

Atorvastatin significantly attenuated the adverse stimulus avoidance learning deficit in the alloxan-induced murine model of diabetes suggesting decreased impairment of memory and cognition.

Diabetes is a metabolic disease that occurs due to insufficient release of insulin from the pancreas or the development of insulin resistance [1]. Diabetes mellitus is a severe condition characterized initially by insulin resistance and hyperinsulinemia; and eventually by glucose intolerance, hyperglycemia, and overt diabetes that cause metabolic and structural changes in several internal organs [2]. The hyperglycemia resulting from diabetes mellitus increases the risk of micro- and macrovascular complications, such as retinopathy, nephropathy, and neuropathy, and causes cardiovascular, cerebrovascular, and peripheral vascular problems [3]. Diabetes mellitus significantly impacts the brain vasculature and central nervous system [4]. Dysfunction in insulin signaling or metabolism is thought to be the reason for several brain pathologies [5]. Loss of memory in diabetes mellitus because of dysregulated glucose–insulin balance has been demonstrated [6]. Type 2 diabetes is associated with an increased risk of Alzheimer disease [7]. Metabolic anomalies in different conditions affect brain–glucose transport and reduce glucose metabolism, resulting in an increased risk of Alzheimer disease [8, 9]. Epidemiological investigations also suggest that diabetes is directly related to Alzheimer disease and could be an important element in Alzheimer disease pathogenesis [10].

Deficiency in attention, reduced psychomotor function, and impaired learning and memory, which are cardinal symptoms of Alzheimer disease, are also seen in patients with type 2 diabetes [11]. Although the primary mechanism remains elusive, constant hyperglycemia, proinflammatory cytokines, and dysregulation in the hypothalamic–hypophysis–adrenal axis is considered collectively responsible for impaired memory and cognitive function [12]. Impaired insulin signaling in the brain due to diabetes mellitus has a role in Alzheimer disease pathogenesis, suggesting that Alzheimer disease is a metabolic brain disease [13]. Insulin sensitivity, amyloid plaque accumulation, neuroinflammation, and oxidative stress are suggested as important pathogenic mechanisms in Alzheimer disease [14]. In prediabetic conditions, dysregulated blood glucose levels pose a risk for memory impairment and cognitive decline, especially in elderly patients. In these patients, diabetes causes harmful inflammation and oxidative stress in the brain [15]. The inflammation has been shown to affect blood vessels and impair brain circulation. These inflammatory effects are thought to be detrimental for the gray matter, which is crucial for decision-making and judgment abilities [16]. Although the relationship between insulin receptors with cognition and memory is extensively investigated, knowledge about the effects of diabetes on memory and cognition is still limited. Alloxan is toxic glucose analog, which modestly prefers the glucose transporter 2 (GLUT2) in the Langerhans cell compared with streptozotocin, commonly used as a pharmacological tool to induced diabetes in rodents [17]. Because alloxan also affects levels of glucose in other sensitive organs such as brain, we choose alloxan to induce glucose-related memory and cognitive impairment.

There is a need for compounds that reduce memory and cognitive decline without affecting other organs. Repurposing compounds that are demonstrated to be clinically safe in other metabolic or vascular disorders is important strategy for developing novel treatment options. As a 3-hydroxy-3-methyl-glutaryl-coenzyme A (HMG-CoA) reductase inhibitor, atorvastatin is an antihyperlipidemic drug currently used in cardiovascular and lipidemia-related diseases [18]. Enhanced antioxidant effects of atorvastatin with concomitant use of high-dose aspirin, metformin, and captopril are demonstrated [19]. Consumption of vegetables, probiotics, and supplements with high antioxidant capacity lower the risk for Alzheimer disease [20]. It is thought that imbalance in metabolism due to diabetes reduces cognitive function, which could be decelerated by improving metabolic balance. Chu et al. [21] showed improved cognitive function after atorvastatin treatment. Kaviani et al. [22] suggested that atorvastatin could be a therapeutic strategy against age-related decline in memory and cognition. However, the effects of long-term atorvastatin use are associated with decreased learning and memory capacity, and recent reports demonstrated that there were no significant effect of statin use on learning and memory function in elderly patients [23]. Samaras et al. [24] investigated long-term statin use, and found that atorvastatin does not significantly affect memory in the elderly, but might have beneficial effects in those with neurodegenerative diseases.

Although studies have investigated possible neuroprotective effects of atorvastatin, knowledge about effect of atorvastatin on diabetes-induced memory loss and the way this effect is associated with blood glucose levels have remained limited to date. In the present study, we used liraglutide, a widely used antidiabetic agent, as a positive control to investigate the possible relationship of diabetes-induced memory decline with blood glucose levels and sought to demonstrate the possible effects of atorvastatin on alloxan-induced hyperglycemia and deficits in their learned response to an adverse stimulus suggesting diabetes-induced memory impairment.

## Methods

### Mice

After obtaining approval from Ondokuz Mayıs University Experimental Animal Research Committee (Hadyek_2020/19), https://ebyssorgu.omu.edu.tr (document No. 9203, verification No. 81DI-HHV0-0ES7), we obtained 35 male BALB/c mice (*Mus musculus*, 30–40 g) raised under specific pathogen free conditions in the Ondokuz Mayıs Vivarium. All experiments were conducted according to the regulations of Ondokuz Mayıs University Experimental Animal Research Committee that at least meet the standards of the National Institutes of Health Guide for the Care and Use of Laboratory Animals (Washington, DC: National Academy Press; 1996). Studies are reported in compliance with the ARRIVE 2.0 guidelines [25]. The mice were maintained in the standard conditions (22 ± 2 °C, 55% humidity, 12–12 night–day cycle, and 3–4 mice per cage) and allowed free access to water and standard laboratory mouse food pellets, except that food pellets were withheld without water restriction for 12 h before alloxan administration, and mice were fed with a 25% glucose solution overnight after alloxan administration to avoid hypoglycemia-induced deaths. Before and every 7 d of the experimental schedule, mice were weighed. A power analysis performed using G*Power software [26] indicated that a sample of 35 mice would be needed to observe the effects with 95% power, which was determined by one-way analysis of variance (ANOVA) between means with an α of 0.05. Assuming possible animal losses based on the mortality rate of the experimental model of diabetes model in previous work would increase the number of mice required to 40. Ultimately, we used 7 mice per group; fortunately, we had no losses and did not have to use extra mice for our experiments. Mice were monitored for behavioral and physical signs of animal welfare [27] and observed for at least 1 h each day by two investigators blinded to treatments, and the mice remained within acceptably humane endpoint criteria with no indication of pain and distress outside of the experimental stimulus and obesity because of treatments. At the end of the experiments, all mice were humanely euthanized using high-dose anesthesia (pentobarbital, 100 mg/kg, i.p.) followed by cervical dislocation.

### Chemicals

Alloxan monohydrate was obtained from Sigma. Atorvastatin (Ator; Sanovel) and liraglutide (Saxenda; Novo Nordisk-Danimarka) were purchased from a local pharmacy in Turkey. All drugs were freshly dissolved in the saline (0.9% NaCl) before administration. Mice were administered 100 μL/kg of each drug at the same time each day.

### Experimental design and alloxan-induced diabetes

Standard mouse food pellets were withheld from the mice for 12 h before alloxan administration. An experimental model of diabetes was induced with 150 mg/kg and 100 mg/kg intraperitoneal (i.p.) alloxan injection at a 48 h interval [28]. Control mice were injected with saline alone as the alloxan vehicle control. Mice were fed with a 25% glucose solution overnight after alloxan injection to avoid hypoglycemia-induced deaths. After 72 h, fasting glucose was measured with the drop of blood collected from the tail vein using a glycosometer (Accu-Chek, Roche). Mice with a blood glucose level of ≥250 mg/dL were accepted as having alloxan-induced diabetes mellitus, and without any specific selection divided into 5 equal groups for treatment: control (n = 7), diabetes mellitus (DM, n = 7, DM + atorvastatin (n = 7), DM + liraglutide (n = 7), and atorvastatin (n = 7). Atorvastatin (10 mg/kg, i.p.) or liraglutide (200 μg/kg, i.p.) was administered daily for 28 d. Atorvastatin and liraglutide doses were selected based on previous reports [29,30,31,32,33]. Liraglutide was used as a positive control for its effect in reducing alloxan-induced hyperglycemia. The 10 mg/kg atorvastatin treatment dose selected in these studies was the lowest neuroprotective dose suggested by the reports [29,30,31,32,33]. All behavioral tests were observed by two investigators blinded to the treatments.

### Oral glucose tolerance and locomotor activity tests

According to methods previously described, an oral glucose tolerance test (OGTT) was performed to investigate glucose homeostasis and the effects of drug treatments on glucose levels [34, 35]. After 12 h fasting without water restriction, mice were treated with atorvastatin, liraglutide, or saline vehicle. After 30 min, 2.0 g/kg glucose was administered, and then blood glucose levels were measured 0.5 h, 1 h, and 2 h later. To evaluate the effects of drug treatments on motor coordination the locomotor activity was measured 24 h after the final drug treatments, after determining the blood glucose levels. The locomotor activity test was conducted using an activity cage (Locomotor Activity Cage; Ugo Basile). The cage consists of horizontal and vertical grids that record and count mouse movements in the selected period. Mice were placed in the cage and their activity was recorded for 10 min, and then the distance that mice traveled was recorded. After the tests, the apparatus was cleaned with super hypochlorous water and 70% ethanol to avoid any bias due to olfactory cues.

### Passive avoidance test

Mice were tested to determine any deficit in their learned avoidance of an adverse stimulus as a model of memory and cognition using a passive avoidance device (Passive Avoidance Apparatus, catalog No. 7550, Ugo Basile). The apparatus consists of an opaque black and an illuminated opaque white plexiglass compartment with an adjoining floor, but separated by a gate. The floor of the apparatus is made of a stainless-steel grid that can conduct electrical current with an indicated program to provide an adverse stimulus to the planter surface of the mouse paws (0.5–1.5 mA, 1–3 s). The test was repeated for 2 d to assess the memory and cognitive capacity of the mice [36, 37]. A mouse was placed in the white compartment and allowed to explore freely for 20 s before opening the gate. Mice that passed through the gate to the black compartment were given an electrical shock to the planter surface of their paws (0.5 mA, 1 s). The time that mice spent in the white compartment before crossing to the dark compartment where they were given the electrical shock was recorded as the initial latency for the first day, and retention period latency for the second day. Additionally, the time that mice spent in the black compartment on the second day was also recorded. This passive avoidance behavior was learned after one trial and normally results in a marked increase in crossover latency. Mice that better “remembered” the adversive stimulus from the first trial spent longer in the white compartment and had a longer retention period latency.

### Statistical analyses

All data were analyzed using IBM SPSS Statistics for Windows (version 21.0). After determination of the data distribution, a one-way ANOVA or a Kruskal–Wallis test was performed. No data were excluded. A general linearized model used to analyze weight data. Multiple comparisons were made by Tukey or Bonferroni post hoc tests. All data are represented as mean ± standard deviation, and 95% confidence interval (CI) and *P* < 0.05 was considered significant.

## Results

### Effects of alloxan and statin and antidiabetic drug treatments on weight, blood glucose levels, and locomotor activity

**Figure 1:**
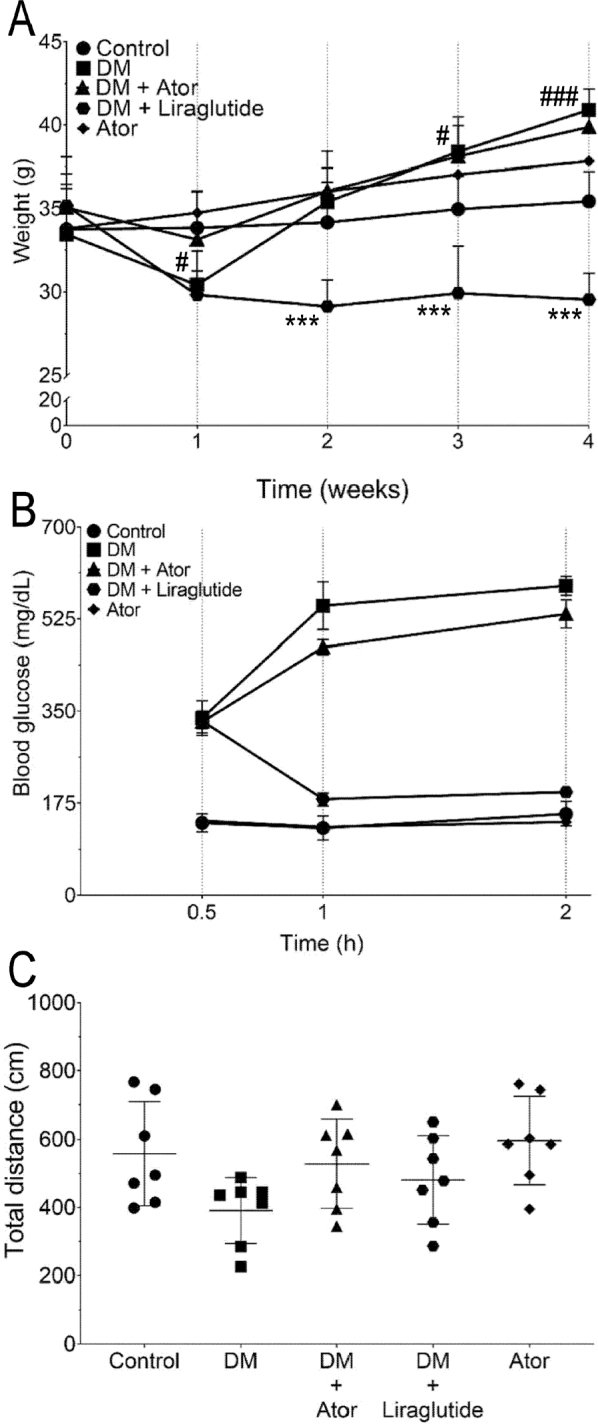
**(A)** Mouse weight throughout the experiment analyzed with a general linearized model. #*P* < 0.05, ###*P* < 0.001 alloxan-induced diabetes vs. vehicle control. ***P < 0.001 liraglutide + alloxan-induced diabetes vs. alloxan-induced diabetes. (**B**) Blood glucose levels after oral glucose administration for each experimental group in the OGTT. (**C**) Locomotor activity for each experimental group. Statistical analyses were conducted using a one-way ANOVA with a Tukey post hoc test. Data are represented as mean ± standard deviation. ANOVA, analysis of variance; Ator, atorvastatin; Control, saline vehicle treatment alone as a negative control; DM, alloxan-induced model of diabetes mellitus; OGTT, oral glucose tolerance test.

Mice in each experimental group were weighed weekly (**Figure 1A**). Time, treatments, and interaction of time and treatments affected mouse weights significantly. Mouse weight was significantly affected by the various treatments (*P* < 0.001 and η_p_^2^ = 0.35 for effect of time, *P* < 0.001 and η_p_^2^ = 0.52 for effect of group, and *P* < 0.001 and η_p_^2^ = 0.44 for effect of interaction of group*time). Alloxan caused significant decrease in mouse weight in the first week, followed by a significant increase in weight in the third and fourth weeks compared with saline vehicle control (**Figure 1A**). Atorvastatin did not affect mouse weights significantly. Liraglutide significantly inhibited the alloxan-induced weight increase (*P* < 0.001, **Figure 1A**). We found no significant difference in the weight of mice between atorvastatin treatment alone and the vehicle control groups (*P* > 0.05, **Figure 1A**). Atorvastatin did not affect blood glucose levels at any time point (*F*_4,10_ = 0.80, *P* = 0.55, **Figure 1B**). Liraglutide decreased blood glucose levels after 0.5 h (*F*_4,10_ = 11.7, *P* < 0.001, **Figure 1B**). Atorvastatin treatment alone did not significantly affect blood glucose levels (*F*_4,10_ = 0.80, *P* > 0.99, **Figure 1B**). We found no significant changes in locomotor activity after diabetes in any drug treatment group (*F*_4,30_ = 2.62, *P* = 0.81, **Figure 1C**).

### Evaluation of initial and retention period latency

**Figure 2:**
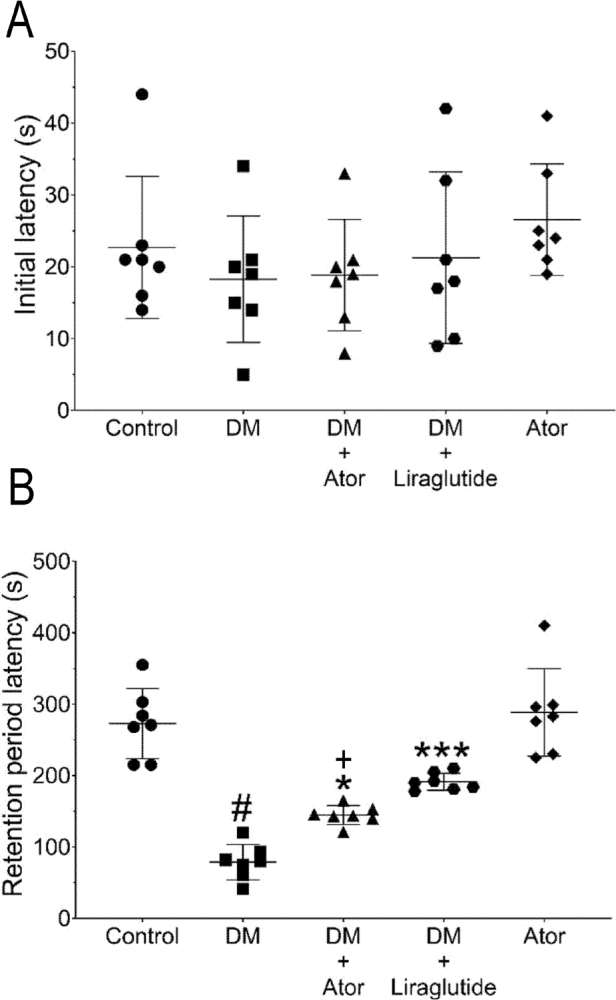
Effect of each drug treatment on (**A**) initial latency and (**B**) retention period latency in the passive avoidance test. There were no significant differences in initial latency between experimental groups. Alloxan-induced diabetes significantly decreased retention period latency. #*P* < 0.05 vs. vehicle control. Atorvastatin significantly attenuated the alloxan-induced diabetes decrease in retention period latency. *P < 0.05 vs. alloxan-induced diabetes; +*P* < 0.05 vs. alloxan-induced diabetes + liraglutide. Liraglutide also significantly attenuated the alloxan-induced diabetes decrease in retention period latency even more than atorvastatin. ***P < 0.001. Symbols represent each data point and the bar indicates mean ± standard deviation. Statistical analyses were performed using a Kruskal–Wallis test. Ator, atorvastatin; Control, saline vehicle treatment alone as a negative control; DM, alloxan-induced model of diabetes mellitus.

We found no significant differences in initial latencies between the treatment groups (*F*_4,30_ = 0.89, *P* = 0.89, **Figure 2A**). On the second day, alloxan-induced diabetes significantly decreased the retention time (79.40 ± 30.25 s) compared with the vehicle control (270.75 ± 66.74 s; *F*_4,30_ = 38.0, *P* < 0.001, **Figure 2B**). Atorvastatin significantly attenuated the decrease (140.30 ± 14.33 s; *F*_4,30_ = 38.0, *P* = 0.02, **Figure 2B**). Liraglutide also significantly attenuated the decrease (197.50 ± 9.73 s; *F*_4,30_ = 38.0, *P* < 0.001, **Figure 2B**). Atorvastatin treatment alone had no significant effect on the latency (288.00 ± 61.30 s) compared with control (270.75 ± 66.74 s; *F*_4,30_ = 38.0, *P* > 0.05, **Figure 2B**).

### Atorvastatin reduced time spent in the dark compartment

**Figure 3:**
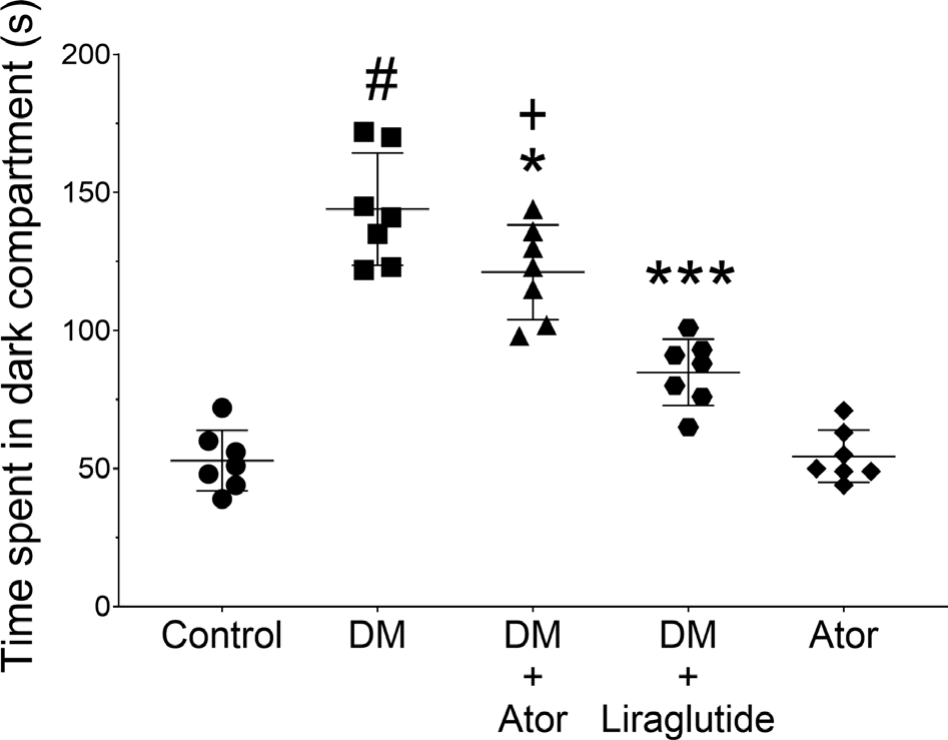
The amount of time spent in the dark compartment by mice in each experimental group. Alloxan-induced diabetes significantly increased the time spent in the dark compartment. #*P* < 0.05 vs. saline vehicle control. Atorvastatin attenuated this increase in mice with alloxan-induced diabetes. *P < 0.05 vs. alloxan-induced diabetes; +*P* < 0.05 vs. alloxan-induced diabetes + liraglutide. Liraglutide significantly attenuated the time mice with alloxan-induced diabetes spent in the dark compartment. ***P < 0.001 vs. alloxan-induced diabetes. Symbols represent each data point and the bars indicate mean ± standard deviation. Statistical analyses were performed using a one-way ANOVA with a Tukey post hoc test. ANOVA, analysis of variance; Ator, atorvastatin; Control, saline vehicle treatment alone as a negative control; DM, alloxan-induced model of diabetes mellitus.

For each experimental group, time spent in the dark compartment was measured (**Figure 3**). Alloxan-induced diabetes significantly increased the time mice spent in the dark compartment (144.83 ± 29.19 s) compared with the vehicle control group (52.83 ± 13.45 s, *F*_4,30_ = 53.9, *P* < 0.001). Atorvastatin significantly decreased the time mice with alloxan-induced diabetes spent in the dark compartment (112.50 ± 13.01 s) compared with mice in the diabetes group without atorvastatin treatment (*F*_4,30_ = 53.9, *P* = 0.046, **Figure 3**). Liraglutide significantly reduced the time mice with alloxan-induced diabetes spent in the dark compartment compared with untreated mice with alloxan-induced diabetes (80.66 ± 11.00 s, *F*_4,30_ = 53.9, *P* < 0.001, **Figure 3**). Atorvastatin treatment alone had no significant effect on the time mice spent in the dark compartment (54.4 ± 9.45 s) compared with the control group (52.83 ± 13.45 s, *F*_4,30_ = 53.9, *P* > 0.05).

## Discussion

The present study suggested that mice with alloxan-induced diabetes had significantly impaired memory and cognitive function. Atorvastatin significantly attenuated the alloxan-induced deficit in learning to avoid an adverse electrical stimulus to the plantar paw, suggesting memory impairment in the mice and this effect of atorvastatin was independent of any effects on blood sugar. We found no significant effect of atorvastatin on the alloxan-induced increase in blood glucose levels. As expected, liraglutide significantly reduced the alloxan-induced increase in blood glucose levels. We found no significant effect of any drug treatment on locomotor activity between the groups and no significant difference in initial latencies between the treatment groups. Therefore, first-day results of the adverse stimulus avoidance test suggest that all mice were similarly adapted (**Figure 2A**). The aversive behavior requires a learned association between a normally neutral environment and the aversive stimulus, and is considered dependent on cognition and memory, which are controlled by the hippocampus, amygdala, and striatal brain structures.

Several studies have demonstrated the neuroprotective and neuroregenerative effects of statins [38, 39]. Li et al. [40] demonstrated protective effects of statins against amyloid β-induced neurotoxicity and neuroprotective effects of atorvastatin against glutamate-induced excitotoxicity in primary cortical neurons [41]. The protective effects of atorvastatin against oxidative insults in the central nervous system indicate possible antioxidant-mediated neuroprotective actions of atorvastatin [40, 41]. These studies investigated the antioxidant effect of acute administration and long-term atorvastatin treatment in vivo and in vitro. Barone et al. [41] demonstrated that long-term high-dose atorvastatin treatment significantly reduced lipid peroxidation, protein oxidation, and nitration in addition to increasing levels of glutathione-S-hydroxylase in beagle brain tissue in a preclinical model of Alzheimer disease. Wasmann et al. [42] found that atorvastatin treatment increased catalase expression and reduced vascular p22phox and nicotinamide adenine dinucleotide (NAD)(P)H oxidase subunit1 (nox1) mRNA expression, which directly indicates a strong antioxidant effect of chronic atorvastatin treatment. Therefore, it appears clear that chronic atorvastatin treatment has antioxidant effect on brain tissue, affecting various molecular pathways, and is thought to be an underlying mechanism behind the neuroprotective effect on Alzheimer-like memory decline. As consistent with these studies, atorvastatin reduced the alloxan-induced deficit in adversive stimulus learning as a model of memory impairment in the present study. Alloxan-induced memory impairment was suggested by the increased time mice treated with alloxan spent in the dark compartment compared with vehicle-treated controls. Considering retention latencies, alloxan-induced hyperglycemia as a model of diabetes mellitus caused memory impairment, as suggested by the retention period latency. Atorvastatin significantly attenuated the alloxan-induced retention period latency suggesting reduced memory impairment (**Figure 2B**). Although atorvastatin did not affect blood glucose levels significantly as liraglutide did, atorvastatin significantly attenuated the alloxan-induced deficit in the behavioral response to the adversive stimulus to almost the same extent as liraglutide, which acted as a positive control to decrease hyperglycemia, suggesting alternative mechanisms.

Our results suggest that atorvastatin, which is a clinically safe drug used to control hypercholesterolemia, may be useful in attenuating diabetes associated memory impairment in those who are treated with this statin. Both conditions are chronically prevalent in the elderly population, and many individuals with both conditions are treated with more than one drug. Single agents that ameliorate multiple clinical conditions are desirable. Nevertheless, it is important to note that prolonged statin use may occasionally result in adverse effects such as liver toxicity and myopathy [43].

A limitation of our study is that the effects of atorvastatin on diabetes-induced memory and cognitive impairment require further investigation to understand the responsible mechanism. Although alloxan produces a useful preclinical murine model of diabetes, conducting experiments in other models such as high-fat diet or streptozotocin-induced diabetes models including insulin treatment may strengthen our findings and further elucidate the potential mechanisms. There is a difference in kinetics for memory loss when mice are trained for passive avoidance, and contextual learning suggests that these two forms of learning use different neural pathways [44]; similarly, there may be differences between long- and short-term memory and we were unable to dissociate them in this study, but they may account for the differences seen between liraglutide and atorvastatin. Results from alternative behavioral assays such as the Morris water maze and Y-maze spatial memory tests would also strengthen our findings. The passive avoidance test and contextual learning tests can be nuanced and may dissect effects in future studies. Metabolic changes and molecular changes, including those of receptors and neurotransmitters and inflammatory mediators in the brain, need to be investigated to elucidate the mechanisms involved. We did not find any effect of atorvastatin on blood glucose levels in this model suggesting its action on the learning deficit is related to other mechanisms, which may include antioxidant effects. As noted for other studies [29], no precise correlation between cholesterol and behavior can be made here as body weight is taken as a surrogate of cholesterol levels. However, the lipid profile is technically difficult to measure because of the small size of the mice and difficulty in collecting adequate blood for measurements.

## Conclusion

In an alloxan-induced model of diabetes mellitus in mice, atorvastatin significantly attenuated decreased avoidance of an adverse electrical stimulus to their plantar paw surface in a murine model of the impaired memory and cognition associated with Alzheimer disease, suggesting that such statins may have a beneficial effect for patients with diabetes and related Alzheimer disease.

## References

[1] Zaccardi F, Webb DR, Yates T, Davies MJ (2016). Pathophysiology of type 1 and type 2 diabetes mellitus: a 90-year perspective. Postgrad Med J.

[2] American Diabetes Association (2017). 2. Classification and diagnosis of diabetes. Diabetes Care.

[3] Zimmet P, Alberti KG, Magliano DJ, Bennett PH (2016). Diabetes mellitus statistics on prevalence and mortality: facts and fallacies. Nat Rev Endocrinol.

[4] Selvarajah D, Tesfaye S (2006). Central nervous system involvement in diabetes mellitus. Curr Diab Rep.

[5] Tumminia A, Vinciguerra F, Parisi M, Frittitta L (2018). Type 2 diabetes mellitus and Alzheimer's disease: role of insulin signalling and therapeutic implications. Int J Mol Sci.

[6] Saedi E, Gheini MR, Faiz F, Arami MA (2016). Diabetes mellitus and cognitive impairments. World J Diabetes.

[7] Pugazhenthi S, Qin L, Reddy PH (2017). Common neurodegenerative pathways in obesity, diabetes, and Alzheimer's disease. Biochim Biophys Acta Mol Basis Dis.

[8] de la Monte SM (2012). Brain insulin resistance and deficiency as therapeutic targets in Alzheimer's disease. Curr Alzheimer Res.

[9] Chornenkyy Y, Wang W-X, Wei A, Nelson PT (2019). Alzheimer's disease and type 2 diabetes mellitus are distinct diseases with potential overlapping metabolic dysfunction upstream of observed cognitive decline. Brain Pathol.

[10] Baglietto-Vargas D, Shi J, Yaeger DM, Ager R, LaFerla FM (2016). Diabetes and Alzheimer's disease crosstalk. Neurosci Biobehav Rev.

[11] Salas IH, De Strooper B (2019). Diabetes and Alzheimer's disease: a link not as simple as it seems. Neurochem Res.

[12] Ryan CM, van Duinkerken E, Rosano C (2016). Neurocognitive consequences of diabetes. Am Psychol.

[13] Gabbouj S, Ryhänen S, Marttinen M, Wittrahm R, Takalo M, Kemppainen S (2019). Altered insulin signaling in Alzheimer's disease brain – special emphasis on PI3K-Akt Pathway. Front Neurosci.

[14] Stanley M, Macauley SL, Holtzman DM (2016). Changes in insulin and insulin signaling in Alzheimer's disease: cause or consequence?. J Exp Med.

[15] Sun Y, Ma C, Sun H, Wang H, Peng W, Zhou Z (2020). Metabolism: a novel shared link between diabetes mellitus and Alzheimer's disease. J Diabetes Res.

[16] Muriach M, Flores-Bellver M, Romero FJ, Barcia JM (2014). Diabetes and the brain: oxidative stress, inflammation, and autophagy. Oxid Med Cell Longev.

[17] Lenzen S (2008). The mechanisms of alloxan- and streptozotocin-induced diabetes. Diabetologia.

[18] Zhou F, Tan Y, Chen X-H, Wu F-L, Yang D-J, Zhang X-W (2018). Atorvastatin improves plaque stability in diabetic atherosclerosis through the RAGE pathway. Eur Rev Med Pharmacol Sci.

[19] Paseban M, Mohebbati R, Niazmand S, Sathyapalan T, Sahebkar A (2019). Comparison of the neuroprotective effects of aspirin, atorvastatin, captopril and metformin in diabetes mellitus. Biomolecules.

[20] Pistollato F, Iglesias RC, Ruiz R, Aparicio S, Crespo J, Lopez LD (2018). Nutritional patterns associated with the maintenance of neurocognitive functions and the risk of dementia and Alzheimer's disease: a focus on human studies. Pharmacol Res.

[21] Chu C-S, Tseng P-T, Stubbs B, Chen T-Y, Tang C-H, Li D-J (2018). Use of statins and the risk of dementia and mild cognitive impairment: a systematic review and meta-analysis. Sci Rep.

[22] Kaviani E, Rahmani M, Kaeidi A, Shamsizadeh A, Allahtavakoli M, Mozafari N, Fatemi I (2017). Protective effect of atorvastatin on D-galactose-induced aging model in mice. Behav Brain Res.

[23] Schultz BG, Patten DK, Berlau DJ (2018). The role of statins in both cognitive impairment and protection against dementia: a tale of two mechanisms. Transl Neurodegener.

[24] Samaras K, Makkar SR, Crawford JD, Kochan NA, Slavin MJ, Wen W (2019). Effects of statins on memory, cognition, and brain volume in the elderly. J Am Coll Cardiol.

[25] Percie du Sert N, Hurst V, Ahluwalia A, Alam S, Avey MT, Baker M (2020). The ARRIVE guidelines 2.0: updated guidelines for reporting animal research. PLoS Biology.

[26] Kang H (2021). Sample size determination and power analysis using the G*Power software. J Educ Eval Health Prof.

[27] Burkholder T, Foltz C, Karlsson E, Linton CG, Smith JM (2012). Health Evaluation of Experimental Laboratory Mice. Curr Protoc Mouse Biol.

[28] Tuorkey MJ (2016). Effects of *Moringa oleifera* aqueous leaf extract in alloxan induced diabetic mice. Interv Med Appl Sci.

[29] Biswas RR, D M C, Rao A S R S, Kadali SRM (2014). Effect of atorvastatin on memory in albino mice. J Clin Diagn Res.

[30] Georgieva-Kotetarova MT, Kostadinova II (2013). Effect of atorvastatin and rosuvastatin on learning and memory in rats with diazepam-induced amnesia. Folia Med (Plovdiv).

[31] Vandresen-Filho S, França LM, Alcantara-Junior J, Nogueira LC, de Brito TM, Lopes L (2015). Statins enhance cognitive performance in object location test in albino Swiss mice: involvement of beta-adrenoceptors. Physiol Behav.

[32] Bugáňová M, Pelantová H, Holubová M, Šedivá B, Maletínská L, Železná B (2017). The effects of liraglutide in mice with diet-induced obesity studied by metabolomics. J Endocrinol.

[33] Knudsen LB (2010). Liraglutide: the therapeutic promise from animal models. Int J Clin Pract Suppl.

[34] Andrikopoulos S, Blair AR, Deluca N, Fam BC, Proietto J (2008). Evaluating the glucose tolerance test in mice. Am J Physiol Endocrinol Metab.

[35] Du L, Liu C, Teng M, Meng Q, Lu J, Zhou Y (2016). Anti-diabetic activities of *Paecilomyces tenuipes* N45 extract in alloxan-induced diabetic mice. Mol Med Rep.

[36] Eagle AL, Wang H, Robison AJ (2016). Sensitive assessment of hippocampal learning using temporally dissociated passive avoidance task. Bio Protoc.

[37] Damián JP, Acosta V, Da Cuña M, Ramírez I, Oddone N, Zambrana A (2014). Effect of resveratrol on behavioral performance of streptozotocin-induced diabetic mice in anxiety tests. Exp Anim.

[38] Bösel J, Gandor F, Harms C, Synowitz M, Harms U, Djoufack PC (2005). Neuroprotective effects of atorvastatin against glutamate-induced excitotoxicity in primary cortical neurones. J Neurochem.

[39] Lu D, Shen L, Mai H, Zang J, Liu Y, Tsang C-K (2019). HMG-CoA reductase inhibitors attenuate neuronal damage by suppressing oxygen glucose deprivation-induced activated microglial cells. Neural Plast.

[40] Li HH, Lin CL, Huang CN (2018). Neuroprotective effects of statins against amyloid b-induced neurotoxicity. Neural Regen Res.

[41] Barone E, Cenini G, Di Domenico F, Martin S, Sultana R, Mancuso C (2011). Long-term high-dose atorvastatin decreases brain oxidative and nitrosative stress in a preclinical model of Alzheimer disease: a novel mechanism of action. Pharmacol Res.

[42] Wassmann S, Laufs U, Muller K, Konkol C, Ahlbory K, Bäumer AT (2002). Cellular antioxidant effects of atorvastatin in vitro and in vivo. Arterioscler Thromb Vasc Biol.

[43] Farmer JA, Torre-Amione G (2000). Comparative tolerability of the HMG-CoA reductase inhibitors. Drug Saf.

[44] Wong ST, Athos J, Figueroa XA, Pineda VV, Schaefer ML, Chavkin CC (1999). Calcium-stimulated adenylyl cyclase activity is critical for hippocampus-dependent long-term memory and late phase LTP. Neuron.

